# Comprehensive Evidence-Based Assessment and Prioritization of Potential Antidiabetic Medicinal Plants: A Case Study from Canadian Eastern James Bay Cree Traditional Medicine

**DOI:** 10.1155/2012/893426

**Published:** 2011-12-20

**Authors:** Pierre S. Haddad, Lina Musallam, Louis C. Martineau, Cory Harris, Louis Lavoie, John T. Arnason, Brian Foster, Steffany Bennett, Timothy Johns, Alain Cuerrier, Emma Coon Come, Rene Coon Come, Josephine Diamond, Louise Etapp, Charlie Etapp, Jimmy George, Charlotte Husky Swallow, Johnny Husky Swallow, Mary Jolly, Andrew Kawapit, Eliza Mamianskum, John Petagumskum, Smalley Petawabano, Laurie Petawabano, Alex Weistche, Alaa Badawi

**Affiliations:** ^1^Canadian Institutes of Health Research Team in Aboriginal Antidiabetic Medicines, Montreal, QC, Canada H3C 317; ^2^Department of Pharmacology, Université de Montréal and Montreal Diabetes Research Center, P.O. Box 6128, Downtown Postal Station, Montreal, QC, Canada H3C 3J7; ^3^School of Dietetics and Human Nutrition and Center for Indigenous Peoples' Nutrition and Environment, McGill University, Sainte-Anne-de-Bellevue, QC, Canada H9X 3V9; ^4^Department of Biology, University of Ottawa, Ottawa, ON, Canada K1N 6N5; ^5^Department of Cellular and Molecular Medicine, University of Ottawa and Therapeutic Products Directorate, Health Canada, Ottawa, ON, Canada K1A 1B6; ^6^Department of Biochemistry, Microbiology, and Immunology, University of Ottawa, Ottawa, ON, Canada K1H 8M5; ^7^Plant Biology Research Institute, Université de Montréal and Montreal Botanical Garden, Montreal, QC, Canada H1X 2B2; ^8^Cree Nation of Mistissini, Eeyou Istchii, QC, Canada GOW 1CO; ^9^The Crees of Waskaganish First Nation, Eeyou Istchii, QC, Canada JON 1RO; ^10^Whapmagoostui First Nation, Eeyou Istchii, QC, Canada JOM 1GO; ^11^Cree Nation of Nemaska, Nemaska, QC, Canada JLY 3BO; ^12^Office of Biotechnology, Genomics, and Population Health, Public Health Agency of Canada, Toronto, ON, Canada M5V 3L7

## Abstract

Canadian Aboriginals, like others globally, suffer from disproportionately high rates of diabetes. A comprehensive evidence-based approach was therefore developed to study potential antidiabetic medicinal plants stemming from Canadian Aboriginal Traditional Medicine to provide culturally adapted complementary and alternative treatment options. Key elements of pathophysiology of diabetes and of related contemporary drug therapy are presented to highlight relevant cellular and molecular targets for medicinal plants. Potential antidiabetic plants were identified using a novel ethnobotanical method based on a set of diabetes symptoms. The most promising species were screened for primary (glucose-lowering) and secondary (toxicity, drug interactions, complications) antidiabetic activity by using a comprehensive platform of *in vitro* cell-based and cell-free bioassays. The most active species were studied further for their mechanism of action and their active principles identified though bioassay-guided fractionation. Biological activity of key species was confirmed in animal models of diabetes. These *in vitro* and *in vivo* findings are the basis for evidence-based prioritization of antidiabetic plants. In parallel, plants were also prioritized by Cree Elders and healers according to their Traditional Medicine paradigm. This case study highlights the convergence of modern science and Traditional Medicine while providing a model that can be adapted to other Aboriginal realities worldwide.

## 1. Background on Diabetes

Diabetes is a chronic metabolic disease that arises from a dysfunction in the body's production of the anabolic hormone insulin, a reduction of the response of peripheral organs to the same hormone, or both [1–3]. There exist two predominant types of the disease; namely, type 1 diabetes (T1D) and type 2 diabetes (T2D) [2, 4, 5]. The former often affects younger individuals and is related to autoimmune responses against insulin or other components related to insulin production that lead to the destruction or severe dysfunction of pancreatic beta cells [2, 4, 5]. Thus, T1D is characterized by insulin insufficiency and is treated by exogenous insulin administration, hence, its former definition as an insulin-dependent type of diabetes. In contrast, the pathophysiological scheme that is generally considered by the contemporary scientific community to explain T2D begins with a gradual attenuation in the response of tissues to insulin, called insulin resistance [2, 4–6]. Pancreatic beta cells that produce the hormone compensate by increasing insulin secretion in response to a given rise in circulating glucose. Eventually, the pancreas decompensates, in good part because of a significant loss in the functional mass of beta cells. This leads to a frank deregulation of blood glucose whereby it remains chronically elevated.

Clinically, the initial phases of the disease are asymptomatic. Indeed, the state of insulin resistance is usually associated with a normal fasting blood glucose (FBG) concentration [7]. However, two elements can help identify this state, namely impaired glucose tolerance (IGT) and hyperinsulinemia [2, 7, 8]. The former can express itself as a blood glucose concentration that reaches beyond 11 mM after a meal or after an oral glucose challenge called oral glucose tolerance test (OGTT). For its part, hyperinsulinemia is related to the aforementioned pancreatic compensation [9]. After pancreatic decompensation, blood glucose remains chronically elevated, as evidenced by fasting hyperglycemia. In fact, it is important at this point to highlight that T2D is a metabolic disease that involves not only the deregulation of glucose homeostasis, but also that of lipids [10]. Indeed, elevated free fatty acids in circulation and the excessive deposition of lipids in abdominal fat or in ectopic sites, such as the skeletal muscle and liver, are recognized as key elements in the development of insulin resistance [11–14].

However, individuals suffering from T2D do not generally succumb to the actual hyperglycemia or dyslipidemia but to their consequences. For instance, it is through a process, coined glucolipotoxicity, that the pancreas is believed to lose its functional mass of beta cells [15]. Elevated blood glucose and lipids also cause micro- and macrovascular lesions. The former affects principally the kidney (diabetic nephropathy), peripheral nerves (diabetic neuropathy), and the retina (diabetic retinopathy). Macrovascular lesions, for their part, lead to cardiovascular disease. T2D is also associated with a state of oxidative stress and chronic low-grade inflammation [16–18]. Finally, several factors, including diabetic neuropathy (loss of sensation and hence increased risk of wounds to the extremities), poor circulation, and a weakened immune response, are at the root of the preponderance of slow-healing wounds in T2D [19, 20]. It is therefore not entirely surprising that T2D is the leading cause of nontraumatic limb amputation, of blindness, of renal hemodialysis, and of cardiovascular disease [7]. Taken altogether, it is these complications of diabetes that cause the high level of morbidity and mortality related to this metabolic disease.

T2D actually represents the pathological endpoint of a cluster of metabolic disturbances that are called metabolic syndrome, syndrome X, or insulin resistance syndrome. There exist several definitions put forth by various national and international agencies but all include a combination of the following factors [7, 21–24]: abdominal obesity, dyslipidemia (notably including increased triglyceridemia, low HDL-cholesterol, and high LDL-cholesterol), IGT, hyperinsulinemia, and hypertension. A cluster of three or more of these factors is necessary for the “diagnosis” of metabolic syndrome. In particular, obesity is the leading risk factor for T2D [25]. With the industrial and food science revolutions of the previous century, most populations around the globe have significantly reduced their physical activity and/or increased their intake of more processed, energy-dense foods. Hence, metabolic diseases have arisen as a result of chronic imbalances between energy intake and energy expenditure. Notwithstanding genetic and other environmental factors (such as stress, pollution, smoking), T2D can rather efficiently be prevented and even treated (notably in its initial stages) by lifestyle interventions [26–28]. However, such changes are difficult to put in place and especially to establish in a persistent manner. Therefore, several therapeutic interventions, mostly centered on pharmaceutical drug therapy, have been developed to prevent or improve the cluster of disorders described above. These are summarized in the following section because they also reflect the relevant targets for medicinal plants stemming from Canadian Aboriginal Traditional Medicine that are the focus of the present paper.

## 2. Contemporary Drug Therapy for T2D

According to the Canadian Diabetes Association Clinical Practice Guidelines [8, 29], a newly diagnosed type 2 diabetic will be prescribed up to five different drugs. These include (1) oral hypoglycemic drugs, alone or in combination, to reduce blood sugar; (2) lipid-lowering drugs, especially to reduce LDL-cholesterol; (3) antihypertensive drugs to reduce blood pressure or prevent hypertension; (4) low-dose aspirin to reduce the risk of thrombosis; (5) insulin, in more advanced stages of the disease. The oral hypoglycemic drugs contain several classes that point to the various targets that can be useful in restoring glucose homeostasis. This is also pertinent in the context of the present case study since these targets also represent the major cell bioassays used to screen medicinal plant preparations for antidiabetic activity.

One of the oldest classes of oral hypoglycemic drugs is represented by the insulin secretagogues, sulfonylureas [30]. These drugs target inward-rectifying potassium channels on the membrane of pancreatic beta cells that are involved in the control of insulin secretion in response to increases in circulating glucose [31, 32]. These drugs have two major limitations. Firstly they confer a risk of hypoglycemia by virtue of the robust insulin secretion they may trigger. Secondly, in view of the aforementioned compensatory mechanisms, sulfonylureas may in fact act to precipitate pancreatic decompensation.

Perhaps the most commonly used oral hypoglycemics are the biguanides, of which metformin is the predominant example [33]. Biguanides were derived from compounds isolated from the French lilac, *Galega officinalis*, a plant long known to be indicated for symptoms of T2D [34, 35]. Metformin has grown to become the drug of first choice in diabetes management worldwide [36], whereas earlier derivatives such as phenformin had to be withdrawn because of life-threatening side effects [37].

It is now known that metformin targets AMP kinase (AMPK), a metabolic master switch enzyme involved in insulin-independent mechanisms that lead to enhanced glucose uptake in skeletal muscle and to reduced hepatic glucose production [38, 39]. Both actions contribute to improving insulin sensitivity and glucose homeostasis (discussed further below).

Thiazolidinediones (TZDs), also known as glitazones, represent a third class of oral hypoglycemic agents that target the nuclear receptor/transcription factor PPAR*γ*, principally in adipose tissue. PPAR*γ* modulates the expression of several genes whose products control both the differentiation of adipocytes and major enzymes involved in lipid homeostasis [40–42]. In experimental and clinical settings, TZDs decrease insulin resistance, in part through their effect of decreasing the ratio of leptin to adiponectin, which are two important adipokines involved in appetite control and insulin sensitivity, respectively [40, 43, 44]. Limitations to their use include hepatic and cardiovascular side effects that have forced the withdrawal of some members of this class from the American and European markets [45]. Less dramatic is the weight gain/water retention that TZDs induce.

Alpha-glucosidase inhibitors act on enzymes of the intestinal epithelial lining involved in the digestion of complex sugars into smaller easily absorbed monosaccharides [46]. They can be used alone or in combination therapy, notably to reduce postprandial hyperglycemia. However, they exhibit dose-dependent gastrointestinal side effects such as flatulence and diarrhea that can limit their use [47, 48].

The newest class of hypoglycemic drugs relates to the incretins, gastrointestinal peptide hormones that act principally on beta pancreatic cells. Incretins, of which GLP-1 and GIP are the predominant species, delay gastric emptying, increase glucose-induced insulin secretion, and stimulate beta cell proliferation [49–51]. The latter effect holds promise to counter the gradually failing pancreatic functional mass characteristic of T2D. Incretins are secreted by intestinal cells and are rapidly degraded by dipeptidylpeptidase IV (DPP-4) enzymes in the blood. Two types of drugs have thus far been developed: the first is degradation-resistant incretin mimetics such as exenatide and the second is DPP-4 inhibitors [51]. Exenatide must be injected subcutaneously and can cause nausea and diarrhea whereas hypersensitivity reactions have been reported for DPP-4 inhibitors.

## 3. Aboriginal Diabetes and Traditional Medicine

Canadian Aboriginals, like several of their counterparts around the globe, exhibit a greater incidence of T2D than their non-Aboriginal peers. This has been related to genetic predisposition and the rapid change in lifestyle [52, 53] moving closer to “western” models and away from traditional behavior, notably traditional food and transportation. In the Cree of Eeyou Istchee (CEI-Eastern James Bay area of Northern Quebec), for instance, the age-adjusted prevalence of T2D is 3- to 5-fold that of non-Aboriginal Quebecers, with an average that reached 29% of the adult population aged 20 years and older in 2009 [54]. This alarming rate of T2D is confounded by the cultural disconnect of the modern pharmaceutical therapies described above. CEI diabetics also suffer from a much greater prevalence of a number of diabetes complications [55–57].

In an effort to identify more culturally relevant approaches to diabetes care, the CIHR Team in Aboriginal Antidiabetic Medicines (CIHR-TAAM) was instated in 2003. Its initial objective was to make the proof of concept that therapeutic approaches based on Cree Traditional Medicine (TM) held promising antidiabetic potential. As with many Aboriginal cosmologies, Cree TM uses a holistic approach whereby physical, mental, emotional, and spiritual components of the “patient” need to be equilibrated. Medicinal plants play an important role in this paradigm, and the CIHR-TAAM has concentrated on providing the scientific evidence base for their antidiabetic potential. Out of respect for the other sacred aspects of Cree healing ways and because the latter are less amenable to conventional scientific study, the CIHR-TAAM has not addressed these issues directly. An ethnobotanical approach was used to identify medicinal plants based on a set of symptoms related to diabetes (discussed further below). Species with the greatest antidiabetic potential were then screened using a comprehensive platform of cell-based and cell-free *in vitro* bioassays as well as *in vivo *animal models of obesity and diabetes, as detailed in the following section.

**Figure 1:**
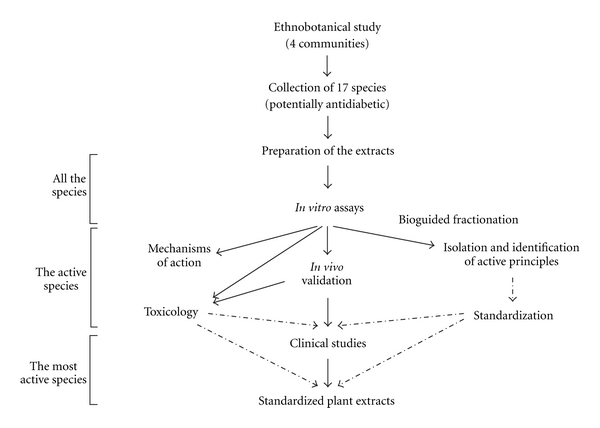
Project flowchart.

Figure 1 shows the flowchart of the project. After ethnobotanical identification and bioassay-based screening (tier 1), the species exhibiting promising biological activities are taken to the next level where more detailed studies are carried out to understand their cellular and molecular mode of action, to identify their active phytochemical principles, and to ascertain their safety and efficacy using *in vivo* animal studies (tier 2). The most active species are then taken to the next level where they are tested in clinical studies with Cree diabetics taking TM alongside the conventional drug therapy (tier 3). Actually, because of the fact that Cree TM has been used for centuries and that several Cree diabetics have decided to call upon Cree Healing Ways, observational clinical studies have begun in parallel with the more detailed laboratory studies mentioned (tier 2). Notwithstanding this particular situation, the following sections will detail the scientific approach taken to provide the evidence base for the antidiabetic activity of the Cree medicinal plants and the protocol taken to prioritize them. Moreover, we undertook to compare this outcome with the prioritization based on Cree TM and cosmology.

## 4. Comprehensive Platform of *In Vitro* Bioassays and *In Vivo* Animal Models of Obesity and T2D

**Figure 2:**
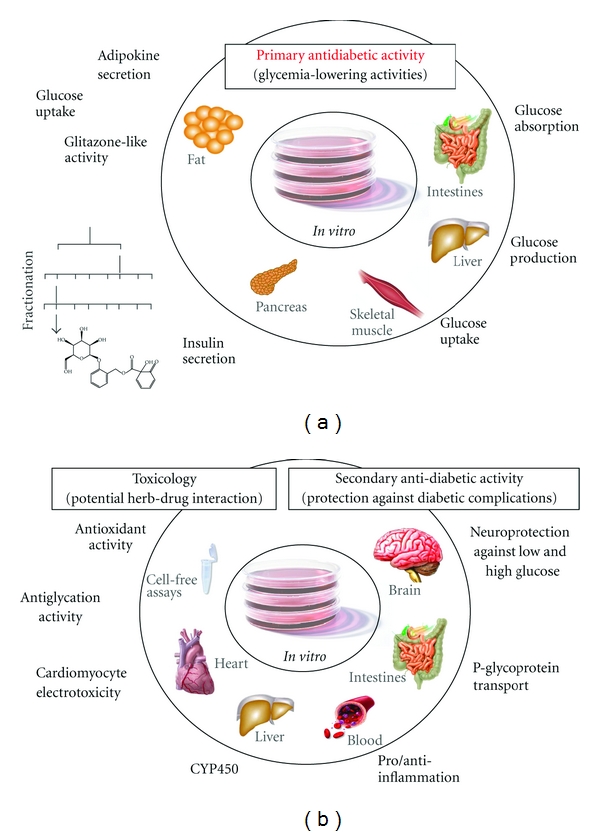
**In Vitro ** screening.

T2D is a multifaceted, multiorgan metabolic disorder, as is indirectly alluded to by the various targets of oral hypoglycemic drugs. Therefore, a platform of *in vitro* bioassays was put in place to screen for primary and secondary antidiabetic biological activities (Figure 2). Primary activities refer to those observed on cells producing (pancreas) or responding (muscle, liver, adipose tissue) to insulin, or involved in glucose absorption from the intestine. What we termed “secondary antidiabetic biological activities” refer to general parameters such as oxidative stress and inflammation, to parameters related to the complications of diabetes, or to the assessment of potential toxicity or herb-drug interactions (Figure 2).

### 4.1. *In Vitro* Screening (Figure 2): Primary Antidiabetic Activities

#### 4.1.1. Assay for Potentiation of Glucose-Stimulated Insulin Secretion

The pancreas is responsible, primarily through insulin secretion from beta cells, to respond to elevations in blood sugar. Pancreatic cell lines such as *β*-TET cells [58] can thus be employed to screen extracts for potentiation of glucose-stimulated insulin secretion (GSIS). These cell lines release insulin in response to physiological glucose concentrations. Changes in secretory properties (basal secretion, GSIS, and shifts in glucose sensitivity) can be detected by measuring insulin released into the medium. Hence, *this bioassay can uncover potential insulin secretagogue actions* reminiscent of sulfonylureas. Also, ^3^H-thymidine incorporation experiments can be used to uncover effects on beta cell proliferation [59]. The latter would indicate a *potential for a given plant extract to favor beta cell regeneration and the replenishment of a functional beta cell mass*. Examples of such effects have been obtained in recent years for antidiabetic plants such as blueberry [60] and Nigella [61]. 

#### 4.1.2. Assay for Potentiation of Glucose Transport

Skeletal muscle is the main site of glucose disposal in human, and approximately 80% of total body glucose uptake occurs in skeletal muscle [62] through insulin- and exercise-sensitive glucose transporters, Glut4. Following exercise or insulin stimulation, Glut4 transporters translocate from intracellular vesicles (basal state) to the cell surface of muscle cells (and, to a lesser extent, of adipose cells) to mediate glucose uptake from the bloodstream. Both the insulin-dependent Akt pathway [10] and insulin-independent exercise pathway that operates through AMPK [63] can modulate Glut4 translocation. The effects of plant extracts on insulin action as well as insulinomimetic activity can thus be assessed by measuring basal- and insulin-stimulated glucose uptake in (1) the C2C12 skeletal muscle cell line and (2) the 3T3-L1 adipocyte cell line. Both types of cell lines have been used as models for insulin-regulated glucose transport for over 15 years [64–68]. In these lines, insulin-stimulated glucose uptake occurs through the GLUT-4 insulin-responsive glucose transporter. In addition, such muscle cell lines also exhibit non-insulin-dependent AMPK-regulated glucose uptake stimulated by metformin, an oral hypoglycemic of the biguanide class. Hence, *these glucose transport assays can identify insulinomimetic, insulin-sensitizing, or insulin-independent antidiabetic potential at the level of skeletal muscle or adipose tissue*.

#### 4.1.3. Assay for Potentiation of Adipogenesis

As mentioned above, thiazolidinediones (glitazones) are a valuable class of antidiabetic drugs that induce an increase in insulin sensitivity by acting on PPAR nuclear receptors and thereby affecting the transcription of a number of genes associated with lipid homeostasis and insulin signal transduction [40]. A widely used screen for glitazone-like activity involves testing for the potentiation of adipogenesis in a differentiating preadipocyte cell line, such as 3T3-L1 cells, as assessed by enhanced accumulation of intracellular triglycerides [69–72]. Rosiglitazone (a reference TZD) serves as a positive control. In the case of positive adipogenic activity, PPAR*γ* agonism can be confirmed by a luciferase gene reporter assay [59]. It is also possible that an inhibitory action be uncovered as happened recently with certain Cree antidiabetic plants [73]. This is not irrelevant since this can represent a potential antiobesity activity as studied in detail recently [74, 75]. Hence, *this bioassay can give insight on the antidiabetic and/or antiobesity potential of plant preparations on adipose tissue*.

#### 4.1.4. Assays for Modulation of Hepatic Glucose Metabolism

The liver is an insulin-responsive tissue that plays a crucial role in the homeostasis of glucose by its ability to control blood sugar level through glucose production or storage, notably in the form of glycogen. This organ also regulates lipid homeostasis through a process implicating key lipogenic enzymes [76]. Finally, the liver plays a prominent role in T2D, notably through weakened insulin-dependent inhibition of glucose production [77, 78]. Hence, hepatocyte cell lines, such as the murine H4IIE and human HepG2 hepatoma-derived cells, can serve to measure the effect of plant preparations on the insulin-dependent and insulin-independent regulations of hepatic glucose metabolism. Glucose-6-phosphatase catalyses a critical step in gluconeogenesis which contributes to enhanced hepatic glucose production in T2D [79, 80]. Inhibition of glucose-6-phosphatase activity thus gives an indication of potential beneficial effect for T2D. Insulin and metformin can be used as positive controls for inhibition of glucose-6-phosphatase activity. Stimulation of glycogen synthesis from glucose is another way to reduce hepatic glucose production [81]. The activity of glycogen synthase, the rate-limiting enzyme of glycogen synthesis, can be measured in hepatic cell lines treated with plant preparations using incorporation of radiolabelled UDP-glucose into glycogen [82]. Treatment with insulin again serves as a positive control for the stimulation of glycogen synthase. Hence, *these hepatic glucose metabolism assays can help identify plant preparations that are likely to reduce hepatic glucose production in vivo, with a corresponding potential to help reduce blood glucose in T2D*.

#### 4.1.5. Assay for Inhibition of Intestinal Glucose Absorption

A number of antidiabetic agents reduce glycaemia by inhibiting digestion and/or absorption of carbohydrates through the gut. These effects are due to either direct interaction with glucose, thereby inhibiting absorption of glucose by enterocytes, direct inhibition of enterocyte SGLT-1 or GLUT-2 transporters, or inhibition of disaccharidases and other enzymes of carbohydrate digestion produced by enterocytes (includes the alpha-glucosidase inhibitor class of oral hypoglycemic drugs discussed above). Evidence already exists for inhibition of digestion and/or absorption of carbohydrates by natural products [22, 83–85]. Furthermore, there is considerable interest in inhibitors of SGLT transporters (intestinal and renal), based on orally active derivatives of phlorizin, as a novel antidiabetic therapy [86–89]. *Intestinal cell lines, such as CaCo-2 cells, can thus serve to probe plant preparations for their potential to inhibit intestinal glucose transport and hence contribute to reducing blood glucose in T2D*. This is the final bioassay used in our platform to test for direct antidiabetic activity.

### 4.2. *In Vitro* Screening (Figure 2): Secondary Antidiabetic Activities

#### 4.2.1. Assay for Cytochrome P450 Inhibition

The cytochrome P450 (CYP) monooxygenase enzyme systems are responsible for a large part of xenobiotic metabolism, notably that of most contemporary pharmaceutical drugs [90]. Plants are known to contain substances that can interfere with the normal activity of several CYP enzymes, notably CYPs 3A4, 2C8, 2C9, and 2D6 [91]. Human recombinant CYP enzymes are commercially available and can be used *in vitro* to assess the level of inhibition by natural products contained in plant preparations, being generally classified as weak (<30%), modest (31–75%), or strong (>75%) inhibition [91]. Hence,* this bioassay will give an initial indication of the risk for herb-drug interactions for a given plant preparation, which represent a major concern in T2D management*.

#### 4.2.2. Assay for Neuroprotective Activity

As mentioned earlier, diabetic neuropathy is one of the major complications of T2D. This results in good part from the damaging impact that chronically elevated blood glucose can have on neurons [92, 93]. This situation can be reproduced *in vitro* by subjecting preneuronal or neuronal cells in culture to hyperglycemic conditions. For instance, preneuronal PC12 cells subjected to high glucose concentrations for 96 h typically exhibit 40–50% cell death, which can be prevented by given medicinal plant preparations [94, 95]. Hence,* neuroprotective activity measured in vitro indicates a good potential for a given medicinal plant to be beneficial against diabetic neuropathy*.

#### 4.2.3. Assay for Antioxidant Activity

As mentioned, T2D is known to be associated with a state of oxidative stress [16, 96]. Secondary metabolites serve several functions in plant physiology such as to protect the plant from damaging environmental factors, notably oxidative stress [16, 96, 97]. Several of these compounds thus exhibit potent antioxidant properties that have been associated with beneficial health outcomes in humans, for instance related to the consumption of fruits and vegetables [98, 99]. There exist several *in vitro*, generally cell-free, tests to determine the antioxidant potential of chemical compounds. Such tests are not physiologically as relevant as those involving intact cells or cell lines. Nonetheless, they provide a rapid and low-cost assessment that is commonly used by academics and industry researchers alike while even beginning to be assimilated by the general population. The oxygen radical absorbance capacity (ORAC) [100] test remains one of the best-known and most commonly used antioxidant tests, despite some limitations. Others such as the DPPH radical scavenging [101] and the thiobarbituric acid reactive substances (TBARS) assays [102] can also be used. *The strong antioxidant activity of a given medicinal plant will offer potential beneficial impacts on T2D*.

#### 4.2.4. Assay for Antiglycation Activity

When blood glucose levels remain chronically elevated, covalent interactions occur between glucose and other blood components, such as proteins. Indeed, one of the proteins subjected to this type of glycation is hemoglobin. In fact, glycated hemoglobin, better known as hemoglobin A1C (Hb-A1C), is used as a reliable index of chronic hyperglycemia [103]. Moreover, reductions in the percentage of Hb-1AC present in the blood of T2D patients are used routinely in the long-term management of glucose homeostasis (CDA clinical practice guidelines; [8, 29]). *In vitro*, it is possible to reproduce the glycation of proteins by incubating a given substrate (e.g., albumin) with high concentrations of glucose for a one-week period. Glycation can then be assessed by fluorometric methods or by Western blot analysis [104]. Hence,* a plant that reduces protein glycation is likely to exert some beneficial actions in the context of T2D*.

#### 4.2.5. Assay for Anti-Inflammatory Activity

Like many chronic diseases, T2D is also associated with chronic low-grade inflammation [17, 18]. This is related, in part, to the increased production of proinflammatory cytokines such as tumor necrosis factor alpha (TNF-alpha). TNF-alpha is also an adipokine produced in quantities in direct relationship to the mass of adipose tissue, hence in an elevated manner in visceral obesity [105]. It is also produced by macrophages when they are activated by microbial components such as lipopolysaccharides (LPSs) [106]. Hence, a standard cell-based bioassay used to assess anti-inflammatory activity measures TNF-alpha (or other inflammatory cytokines) released by LPS-activated macrophages in culture put in contact with the tested agent, in the present case, a given medicinal plant preparation. Hence,* if a medicinal plant exhibits significant anti-inflammatory activity, this will represent a potential benefit in the context of T2D*.

### 4.3. *In Vivo* Screening (Figures 3 and 4)

**Figure 3:**
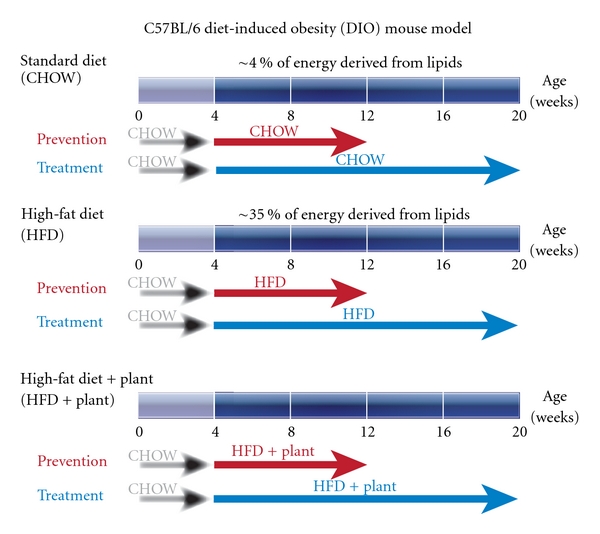
**In Vivo ** screening.

**Figure 4:**
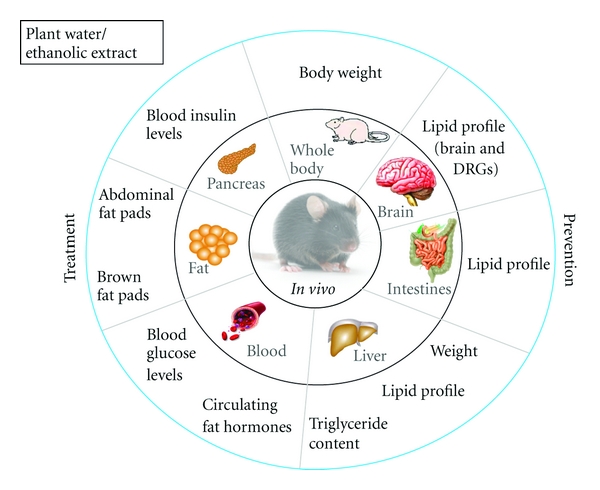
**In Vivo ** screening.

Biological effects of plant preparations in primary antidiabetic bioassays should hold potential to mediate a reduction in blood glucose *in vivo*. A number of animal models of obesity and diabetes exist to evaluate the therapeutic potential of medicinal plants. Some rely on genetic defects that predispose animals to obesity (e.g., *Ob/Ob* mice) or diabetes (e.g., Zucker diabetic fatty (ZDF) rats) or chemicals that induce such disorders (e.g., streptozotocin or alloxan). Others use dietary interventions that cause metabolic disturbances in animals that resemble the spectrum spanning from insulin resistance to T2D. The CIHR-TAAM has experimented with many of these models [107, 108], and the diet-induced obesity (DIO) model in mice has been found to provide stable and reliable results. This model is illustrated in Figures 3 and 4 and is briefly described further below.

#### 4.3.1. Diet-Induced Obesity Mouse Model

The male C57BL/6J mouse has been considered as a gold standard to generate the diet-induced obesity (DIO) animal model [109]. This mouse species develops an obesity phenotype only when given free access to a high-fat diet whereas individuals remain normal when fed a low-fat diet (Figure 3). The weight gain in C57BL/6J mice on the high-fat diet results from a combination of increased energy intake and decreased metabolic rate [110, 111]. In addition to the typical feature of obesity, C57BL/6J mice on the high-fat diet also develop insulin resistance, impaired glucose tolerance, mild to moderate hyperglycemia, dyslipidemia, hypoadiponectinemia, leptin resistance/hyperleptinemia, and hypertension (Figure 4). DIO mice suffer from islet dysfunction, decreased uncoupling protein-2 (UCP2) expression, and downregulated *β*_3_-adrenegic receptor expression and function [112, 113]. The course of diabetes development and the interaction of nutritional components with genetic variables in C57BL/6J mouse closely mimic the progression of human diabetes [114]. Numerous studies have demonstrated that the C57BL/6J DIO mouse is a suitable animal model for examining novel therapeutic interventions and how diverse antidiabetic drugs exert *in vivo* efficacy [115, 116]. Therefore, the DIO mouse model can be used to study the efficacy and systemic mode of action of medicinal plants on the prediabetic and early stages of obese T2D. Plant preparations can be administered to animals in various ways. Intravenous or intraperitoneal injections are sometimes used, but these do not reflect the oral administration characteristic of the vast majority of traditional preparations. Gastric gavage using blunt needles is an efficient way to deliver plant preparations while respecting precise dosages and administration regimens. However, in small animals like mice, the risk of gastric puncture significantly increases with the frequency and duration of plant administration. Moreover, the usual dead volume of gavage needles can become a barrier, especially when dealing with more purified preparations where only small quantities are often available. It is therefore also common for plant preparations to be incorporated into either the drinking water or the feed of laboratory animals. The advantages are that plant preparations can be rather easily and homogeneously integrated into these matrices. On the other hand, the dosing requires careful assessment of the amounts of food and water that are consumed; attention also needs to be paid to avoid spillage or wastage of food or water in order to prevent errors in dosing. Nevertheless, such approaches appropriately mimic oral administration, albeit less so regarding dosing frequency, especially in continuous feeders. Oral feeding/drinking strategies also imply carrying out initial dosing studies to find the optimal range of concentrations of plant preparations. It is common to use a dose of 100 mg/kg body weight as a reference starting point for crude plat extracts, although this does not bear a direct relationship with a human therapeutic dose. In this instance, doses of plant preparations in animals, as is also the case for conventional drugs, cannot easily be translated into human equivalents.

## 5. Prioritizing Promising Antidiabetic Plants Based on Traditional and Scientific Evidence

The first level of prioritization actually relates to the knowledge of Cree elders and healers. Indeed, the medicinal plants subjected to scientific testing need to be identified through their use in TM. Ethnobotany is the science that studies the use of plants and plant-related materials by human populations for several functions, a major one being health and wellbeing. Several methods exist to study the traditional knowledge of human populations, informant consensus being a common one [117, 118]. However, such approaches are based on the assumptions that the healers know the ailment/disease and that they use plants for such conditions. Since T2D was very rare in Canadian Aboriginal populations even as recently as 50–60 years ago, a novel ethnobotanical approach was developed by the CIHR-TAAM [117, 118]. This method relies on a set of 15 symptoms related to T2D. Traditional knowledge holders are asked about which plant they would use for a given symptom. Medicinal plant species are then ranked, taking into account (1) the number of healers that mention a given plant and (2) the number of symptoms for which the given plant is used. Results are also weighed according to the importance/relevance of each symptom to T2D, since some (e.g., slow-healing wounds) are quite specific to T2D while others (e.g., diarrhea) are not. The result of the algorithm is called a syndromic importance value (SIV) that essentially represents the antidiabetic potential of a given plant. Seventeen such plants were prioritized in this way for scientific assessment by the CIHR-TAAM after interviewing 104 Cree elders/healers in four different communities of CEI.

Secondly, from the scientific point of view, results from the *in vitro* bioassay-based screening and the *in vivo* assessment of safety and efficacy can be used to rank plants according to their potential usefulness in the context of T2D.  Table 1 presents a clear example of such a prioritization exercise using Boreal forest plants from the CIHR-TAAM project. Plant names have been codified in order to respect and preserve the intellectual property rights related to Aboriginal TM.

*In vivo* animal models of obesity and T2D integrate all the pharmacodynamic (tissue, cellular, and molecular targets) and pharmacokinetic (absorption, distribution, metabolism, excretion) elements that will respond to, and impact on, putative antidiabetic plant preparations. Hence, it is logical to put a greater priority on positive results obtained *in vivo* in comparison to those obtained using *in vitro* bioassays. This explains why results obtained in animals with the various Boreal forest plants come at the top of the prioritization list in Table 1. In view of the pathophysiology of T2D discussed above, the maintenance of normal blood glucose levels is the primary goal of T2D therapy. Hence, the top outcome to prioritize in order to identify a plant as potentially antidiabetic relates to its ability to reduce blood sugar in a diabetic animal. As also mentioned in the background section, obesity is the single most significant determinant of the risk for T2D. Hence, the second parameter chosen to rank plants according to their antidiabetic potential relates to a given plant's ability to reduce body weight. Thirdly, as discussed earlier, the ectopic accumulation of fat in the liver plays a significant role in the pathophysiology of insulin resistance and T2D, hence the inclusion of a given plant's capacity to reduce hepatic steatosis in the ranking protocol.

Next, results of *in vitro* bioassays of direct antidiabetic biological activity can help rank plants according to their target tissue and biological action. As mentioned, skeletal muscle is the single most important tissue for glucose disposal in mammals. Uptake of glucose into muscle cells in culture was thus chosen as the first *in vitro* parameter to use in the prioritization scheme. Coming in a close second is the potential for plant preparations to reduce hepatic glucose production. That is why the combined effect of a given plant to inhibit glucose-6-phosphatase and to stimulate glycogen synthase (two key enzymes countering the production of glucose by the liver) was chosen to rank antidiabetic plants. Next, favoring the differentiation of adipocytes is a trait that was sought in medicinal plant preparations because of the associated capacity to store both glucose and free fatty acids where they are best preserved, to maintain normal glycaemia and reduce insulin resistance. Finally, inhibition of intestinal glucose transport *in vitro* holds promise for potential beneficial action to reduce glucose absorption.

Finally, what were coined “secondary antidiabetic biological activities” may also be considered when prioritizing medicinal plant species. Both Cree Elders and clinical endocrinologists involved in the clinical studies of the CIHR-TAAM have voiced their concern about using Cree TM alongside modern pharmaceuticals in the context of T2D. Therefore, the potential for plants to affect the metabolism of xenobiotics, notably drugs, must be taken into consideration since TM is often taken alongside conventional antidiabetic drugs. Inhibitory activity against recombinant human cytochromes P450 was thus included as an important parameter, with the least inhibition being the desired characteristic of prioritized medicinal plants. Cytoprotective action, notably towards cells of neuronal phenotype, is also important to consider in view of the T2D-associated complication, diabetic neuropathy. Neuroprotective activity was therefore measured and included as a parameter to rank antidiabetic plants. Last but not least, antioxidant, antiglycation, and anti-inflammatory activities were selected in view of the known involvement of oxidative stress and low-grade chronic inflammation in T2D.

As can be appreciated from Table 1, several plants of the Boreal forest that were identified through the novel ethnobotanical approach of the CIHR-TAAM exhibit very interesting combinations of primary and secondary antidiabetic activities. This confirms the validity of the ethnobotanical method and especially the great wisdom/value of Aboriginal healers and their traditional knowledge. However, the prioritization scheme presented in Table 1 is based solely on evidence-based scientific studies. It was therefore interesting and important to compare the results of this prioritization exercise with the perceptions of Cree Healers within their Aboriginal worldview and TM paradigm. When asked to prioritize plants from their TM perspective and to justify their choice, Cree healers spoke of their respect for a given plant and shared healing experiences that they had or witnessed with the same plant. Their prioritization was thus much more grounded in the spiritual realm, in personal experience, and natural laws. Notwithstanding how different the two worldviews can be, the most striking outcome was that the majority of plants ranking among the most promising antidiabetic plants, as prioritized by the scientific approach, were the same as those held to the greatest esteem by Cree healers, 4 out of 6. This speaks highly for a convergence of health parameters across cultural barriers. Instead of “validating” Cree TM (which may be seen as carrying negative connotations, especially from the Aboriginal TM perspective), the work of the CIHR-TAAM may rather find its value and usefulness in “translating” Aboriginal traditional knowledge into the language of evidence-based science that health authorities can better appreciate.

## Figures and Tables

**Table 1:** Prioritization of Boreal forest medicinal plants according to **in vivo ** and **in vitro ** antidiabetic activity.

			Plant identification					
			A	B	C	D	E	F
Elders' ranking				3	2	1		5
Biological activities related to primary actions against diabetes	Animals	Decrease blood glucose	***☺***	***☺***	***☺***	***☺***	***☺***	**?**
		Reduce body weight	***☺***	***⊗***	***⊗***	***⊗***	***⊗***	**?**
		Reduce fatty liver	***☺***	***☺***	***☺***	***☺***	**?**	**?**
	Cells	Move glucose into muscle cells	***⊗***	***☺***	***☺***	***☺***	***☺***	***☺***
		Reduce glucose produced by liver cells	***☺***	***☺***	***☺***	***☺***	Moderate	Moderate
		Favour good fat	***⊗***	***☺***	***☺***	***☺***	Moderate	***⊗***
		Decrease glucose absorbed from food	***⊗***	***☺***	***☺***	Moderate	***⊗***	***☺***

Biological activities related to diabetes complications	Cell free	Safe to mix with drugs	***☺***	Moderate	***☺***	Moderate	Moderate	***☺***
		Fight bad oxygen, bad glucose	***⊗***	***⊗***	***⊗***	***⊗***	***⊗***	***⊗***
	Cells	Fight inflammation	***☺***	***⊗***	***☺***	***☺***	***⊗***	***☺***
		Protect nerves	*☺*	***⊗***	***⊗***	***⊗***	*☺*	*☺*

Smiley faces: positive effect; ***⊗***: no effect; ?: yet undetermined. The data that forms the basis of this table has been collated from several studies that have already been published by our team [73–75, 91, 94, 95, 104, 108, 117, 119, 120] as well as data (especially from *in vivo* studies) that have not yet been published (several currently under review). The names of plants must thus remain undisclosed to protect both the traditional knowledge shared by Cree Elders and the unpublished data.
